# Establishing a Collaborative Genomic Repository for Adult Burn Survivors: A Burn Model System Feasibility Study

**DOI:** 10.3390/ebj5040034

**Published:** 2024-11-06

**Authors:** Stephen Sibbett, Jamie Oh, Gretchen Carrougher, Lara Muffley, Nathaniel Ashford, Maiya Pacleb, Samuel Mandell, Jeffrey Schneider, Steven Wolf, Barclay Stewart, Nicole S. Gibran

**Affiliations:** 1 Department of Surgery, University of Washington, Seattle, WA 98195, USAnicoleg@uw.edu (N.S.G.); 2 Department of Genome Sciences, University of Washington, Seattle, WA 98195, USA; 3 Parkland Regional Burn Center, Department of Surgery, University of Texas Southwestern, Dallas, TX 75235, USA; 4 Rehabilitation Outcomes Center at Spaulding Rehabilitation Hospital, Department of Physical Medicine and Rehabilitation, Harvard Medical School, Boston, MA 02129, USA; 5 Department of Surgery, University of Texas Medical Branch, Galveston, TX 77555, USA

**Keywords:** genomics, worrier/warrior gene, precision medicine, multicenter database

## Abstract

In this study, we aimed to integrate a genetic repository with an existing longitudinal national burn database. We set out two primary objectives, namely (1) to develop standard operating procedures for genetic sample collection and storage, DNA isolation, and data integration into an existing multicenter database; and (2) to demonstrate the feasibility of correlating genetic variation to functional outcomes in a pilot study, using the catechol-O-methyltransferase (COMT) gene. Dubbed the worrier/warrior gene, COMT variants have been associated with varying phenotypes of post-traumatic stress, wellbeing, and resilience. Between August 2018 and July 2020, COMT variants were identified for 111 participants from three sites and correlated with their outcome data. We found no association between COMT variants and functional outcomes, likely due to the inadequate sample size. We also asked all potential participants why they consented to or refused genetic analysis. A thematic analysis of responses revealed altruism and personal interest/enthusiasm in the study as top reasons for consenting. Privacy concerns were the most common reason for refusal. In conclusion, we successfully developed standard operating procedures for genetic sample collection and storage, DNA isolation, and data integration into an existing database, and we demonstrated the feasibility of conducting a multicenter collaborative study using a centralized lab location.

## 1. Introduction

Advances in burn care over time have led to decreased mortality worldwide [1]. Although this trend is encouraging, recovery often challenges people with burn injury across their lifetimes [2]. After the initial injury, complex, multidisciplinary, and long-term care is necessary to ensure proper wound healing, mitigate scarring, and maximize functional recovery. Physical consequences include pain [3,4], itching [4,5], sleep issues [6], and loss of physical function [7]. Acute and post-traumatic stress, depression, anxiety, and other psychological comorbidities are also common [8,9]. Databases have played a key role in the advancement of longitudinal burn care needs and recovery, recommending health system functions and service delivery and identifying potential quality improvement interventions [10,11]. These resources collect data ranging from demographic information, injury characteristics, hospitalization, care utilization data to functional outcomes and health-related quality of life (HRQoL) measures. The utilization of these databases has led to improvements in our understanding of burn injury, such as the recognition of acute burn injury as a chronic condition [12,13,14].

As comprehensive as these databases are, opportunity remains for expansion with the identification of both modifiable and non-modifiable predictors of key recovery and quality of life outcomes. One such area is the inclusion of genetic data. Precision medicine, which involves the use of genetic data to maximize an individual patient’s responses to potential therapies, has become increasingly prevalent across medical specialties [15]. The use of human epidermal growth factor receptor 2 (HER-2) status in breast care management is often cited as a successful example of precision medicine in practice [16,17]. Previous work conducted by our group identified an association between melanocortin 1 receptor R163Q polymorphism and hypertrophic scarring among people with burn injury [18]. We hypothesize that genetic factors influence physical and psychological recovery after burn injury and might serve as targets for precision medicine in the future.

One potential target is the catechol-O-methyl transferase (COMT) gene, which encodes an enzyme responsible for the inactivation/degradation of the catecholamine neurotransmitters dopamine, epinephrine, and norepinephrine. The Val158Met (rs4680) variant in this gene has been termed the “worrier/warrior” allele because of the strong association between allelic variation and dopamine levels [19], pain thresholds and stress resiliency [20], and executive cognitive functioning [21]. Individuals with two A alleles are considered “worriers” and those with two G alleles “warriors”. Depending on the COMT variant, individuals have varying phenotypes of post-traumatic stress disorder [22], wellbeing [23], and functional outcomes following mild traumatic brain injury in veterans [21,24,25,26].

Building on our experience with the correlation of genetic variation with scarring and itching [18], we aimed to integrate a genetic repository with an existing longitudinal national burn database centered on recovery, rehabilitation, and quality of life after burn injury. We set out two primary objectives in this feasibility study, which were (1) to develop standard operating procedures for genetic sample collection and storage, DNA isolation, and data integration into an existing multicenter database; and (2) to demonstrate the feasibility of correlating genetic variation to functional outcomes in a pilot study, using the Val158Met polymorphism in the COMT gene as our gene of interest.

## 2. Materials and Methods

Funded by the National Institute for Disability, Independent Living and Rehabilitation Research, the Burn Model System (BMS) multicenter genetics module was approved by the BMS program directors as part of the overarching BMS program. The BMS National Database collects longitudinal data from people with burn injury who meet the following criteria:

- Surgical intervention to achieve wound closure during the index (acute) hospitalization;

AND

- Those 65 years of age or older with a total body surface (TBSA) equal or greater than 10%;

OR

- Those 0–64 years of age with a TBSA equal or greater than 20%; OR
- All ages with an electrical or lightning burn injury; OR
- All ages with a burn affecting any area of the face, hands, or feet.

### 2.1. Collection of Genetic Data

Buccal swabs were collected from enrolled BMS participants with institution-specific ethical approval at each collaborating BMS site (Sites A, B, C). Swab kits consisting of buccal swabs (Puritan PurFlock Ultra), sample identification labels, postage paid-return envelopes, and human specimen labels for the return envelopes were assembled by Site A and sent to collaborating sites. An overview of our proposed standard operating procedure is provided as follows:

1. Buccal swab sample collection.
2. Sample transport to BMS laboratory.
3. Sample storage at −20 °C.
4. DNA isolation and quality assessment.
5. DNA storage at −80 °C.
6. Downstream genetic applications (i.e., COMT SNP analysis).

Adult participants in the BMS National Database at all timepoints were asked to participate in the study by providing a buccal swab sample. Swabs collected at Site A were immediately stored at −20° in the Site A translational laboratory. Collaborating sites sent their swabs to Site A via the United States Postal Service (USPS). Participants who consented to swab collection but were unable to provide a sample on site sent swabs directly (participant-to-lab) to the BMS lab (Site A only). Swabs were stored at −20 °C until DNA isolation; arrival and processing dates and times were documented.

DNA was isolated from swabs using the MagMAX™ DNA Multi-Sample Ultra 2.0 Kit (Invitrogen™) and processed with the KingFisher™ Duo Prime Purification System (Thermo Scientific™). We used a modified version of the KingFisher™ Duo’s MagMAX™ DNA Blood Buccal program with an additional wash step to maximize DNA isolation. DNA quantity and quality were assessed using the NanoDrop™ 2000 Spectrophotometer (Thermo Fisher Scientific). Isolated DNA was aliquoted to maximize future use and stored at –80 °C. Data were uploaded to a secure REDCap database linked to the master REDCap-based BMS National Database. Processed swabs were submerged in a 10% bleach solution to destroy remnant DNA and disposed of following institutional procedures for biohazardous waste disposal.

COMT SNPs were determined using a TaqMan™ SNP Genotyping Assay with primer (Thermo Fisher Scientific™ cat. C__25746809_50, Waltham, MA, USA), TaqMan™ Genotyping Master Mix (Applied Biosystems™, Waltham, MA, USA), and QuantStudio™ 7 Flex Real-Time PCR System (Applied Biosystems™, Waltham, MA, USA). PCR was repeated once for failed reactions.

- Primer:

CCAGCGGATGGTGGATTTCGCTGGC[A/G]TGAAGGACAAGGTGTGCATGCCTGA.

### 2.2. Feasibility and Implementation Improvements

Because this was a feasibility project to demonstrate that genetic data could be added to a longitudinal outcomes database, we repeatedly evaluated steps of the process to troubleshoot pitfalls. Multiple interventions were implemented throughout the study to increase participation and DNA quality and yield, particularly with swabs returned by mail. An infographic with instructions for proper sample collection and mailing procedures was included with the swab and return envelope. To ensure adequate DNA collection from mailed-in samples, we created a YouTube video at https://www.youtube.com/watch?v=oSvMEyslWtY (uploaded 29 March 2019) as a web-based tutorial for participants and research coordinators to emphasize techniques for optimal buccal smear collection. COMT SNPs for 111 samples collected between August 2018 and July 2020 were correlated with corresponding BMS PROMIS-29, Veterans RAND-12 (VR-12), Post-Traumatic Growth Inventory (PTGI), and pain interference data at hospital discharge and six months after injury.

### 2.3. Data Analysis

We used Kruskal–Wallis and chi-square tests to determine associations between COMT SNPs and patient demographics, burn descriptors, and functional outcomes. Our variables of interest are described below.

The PROMIS^®^ instruments were developed as non-disease-specific measures of health-related domains. Each PROMIS-29 measure is scored with a z-score of 50, representing the population mean, a standard deviation of 10, and higher scores indicating higher levels of a particular measure [27]. The non-proprietary Veterans RAND-12 is a brief self-report survey of patients’ views of their own health and is reported as a z-score, where 50 is the population mean and 10 represents one standard deviation [28]. Higher scores indicate a more positive patient-reported view of their health. The Post-Traumatic Growth Inventory (PTGI) was developed to assess post-traumatic growth and self-improvement, with higher scores indicating greater growth [29]. Post-traumatic growth generally is a positive psychological change experienced because of struggling with highly challenging and highly stressful life circumstances (e.g., major burn injury).

All potential participants at Site A were asked to write up to three reasons why they consented or declined to provide a swab sample. Responses were sorted into themes using content analysis software (Dedoose Version 9.0.107, Los Angeles, CA, USA: SocioCultural Research Consultants, LLC). Differences in codes and code hierarchy were discussed among authors until consensus was reached. Codes were revised and summarized with exemplary quotations.

## 3. Results

### 3.1. Feasibility

Between August 2018 and July 2020, 126 buccal swabs were collected by three BMS sites. All mailed swabs were received using the USPS, indicating feasibility of both the multicenter and direct participant-to-lab collection process. DNA isolation (Table 1) yielded a total volume of 50 μL for each swab, with a median concentration of 67.05 ng/μL DNA per sample. Swabs from Site A contained more DNA than the other sites (103.3 compared to 30.2 and 28.5 ng/μL). All the USPS-mailed swabs were considered “mailed in”, including direct participant-to-lab and Site B and C samples. More DNA was isolated from swabs collected at Site A (169.7 ng/μL) compared to the mailed-in swabs (33.45 ng/μL).

The distribution of COMT SNPs via PCR was consistent across sites and with previous population studies (Table 2), with the heterozygous AG variant being the most frequent (41%), followed by the AA “worrier” variant (31%) and the GG “warrior” variant (28%) [30,31]. Samples with higher DNA concentrations were more likely to be associated with successful PCR reactions (79.5 compared to 8.2 ng/μL median isolated DNA, Table 1); successful reactions occurred in samples with as little isolated DNA as 4.02 ng/μL (Table 1).

An initial concern about such a translational project included the cost to the central lab site (Site A) as well as each participating site. Overall, financial constraints had a negligible impact on the execution of this Burn Model System genetics project. The Site A translational laboratory included existing equipment appropriate for DNA isolation and sufficient freezer storage space. As such, laboratory startup expenses were minimal. Accounting for consumable supplies, shipping and research costs, and coordinator effort, it cost less than $5 per sample to generate such a DNA repository. Up to 12 swabs can be processed at a time, which took about 2.5 h for trained research staff.

### 3.2. Cohort and Correlation

Our study population predominantly consisted of white (n = 97, 87%) and non-Hispanic (n = 89, 80%) males (n = 75, 68%). There was no evidence for an association between the COMT genotype and demographic or injury characteristics (Table 3). Table 4 shows COMT genotype correlation with participants’ outcomes. There was no difference among COMT variants in VR-12 scores. VR-12 mental component scores (MCSs) were above the population mean at baseline and remained higher at the six-month follow-up. VR-12 physical component scores (PCSs) were lower at the six-month follow-up than they were pre-injury (measured by recall at discharge). There was no statistical difference between COMT variants and either PROMIS^®^-29 or VR-12 pain interference measures.

### 3.3. Thematic Analysis

A thematic analysis on BMS National Database participant responses about why or why not they consented (Table 5) identified four themes among 80 responses, namely altruism, relationship with burn center staff, interest/enthusiasm, and ease of participation. Most responses fit into altruism and interest/enthusiasm (65 total). Six responses explicitly cited a relationship with burn center staff as the reason they chose to participate. Nine responses noted that swab collection was easy or was not perceived as causing excessive burden. Some responses were assigned to multiple themes. For example, the response “I want to help others; you have done much for me” was categorized into both “altruism” and “relationship with burn center staff”. For declining participants, we identified three themes in 26 responses. Twenty-three of these responses alluded to privacy concerns and a lack of interest in participation. Three responses were categorized as “other”; one patient was not interested without knowledge of their own results, another described the study as “weird”, and the third cited worry about their susceptibility to SARS-CoV-2.

## 4. Discussion

With continuous attention to and resolution of operational barriers, we have demonstrated feasibility of incorporating data from high-quality genetic material into a multicenter longitudinal outcomes database. The substantial difference in the amount of DNA collected onsite at Site A compared to mailed samples (Table 1) was most likely due to the suboptimal collection technique. This difference ultimately did not impact SNP analysis, as just 60 ng of total DNA was required per sample. Most samples yielded much more than 60 ng DNA, allowing for a potential evaluation of other SNP targets. In future iterations of a genetic repository, it might be most efficient to sequence the entire genome for the evaluation of multiple target genes. Protocols from other genetic studies and commercial laboratory services recommend a minimum of 200 ng of genomic DNA at a concentration greater than 30 ng/μL for both GWAS and whole-genome sequencing [32,33,34]. Most of our samples meet these requirements. Performing whole genome sequencing would provide several benefits. Samples would only need to be processed once, eliminating concerns for DNA quality with multiple GWAS studies requiring multiple freeze–thaw cycles. The storage of genome data would also provide more security than physically storing isolated genetic material. As potential target genes are identified in the future, having genome data collected over years could be easily targeted with adequate sample sizes.

Although we recognized that a population genetic analysis requires many more samples to demonstrate associations, our successful COMT genotyping in 111 of 126 samples (88%) with comparable allelic distribution to other studies [30,31] confirmed that genetic data can be merged with and analyzed alongside longitudinal HRQoL data. A lack of strong evidence for correlation between the COMT genotype and demographic information, injury characteristics, or functional recovery measures is due in part to insufficient sample numbers. The inclusion of 50 subjects for each COMT variant would be required to detect a large effect size with a one-sided significance level of 0.05. Even without sufficient power, our data are interesting in that the pre-injury VR-12 MCSs were nearly a full standard deviation above the US population mean and remained high at the 6-month follow-up, indicating greater satisfaction with mental health compared to the general US population. As anticipated, six months after injury, VR-12 physical component scores (PCSs) were below the mean.

The use of qualitative data analysis provided important perspectives regarding the participation in genomic research [35,36]. Among consenting participants, nearly half of the responses mentioned altruism as a primary reason for contributing buccal swabs. The potential to aid future burn survivors through research participation could provide a sense of purpose and aid psychological recovery [37,38]. A sense of potential to help others might also relate to our study population’s VR-12 mental component scores (MCSs) being higher than the US population mean six months after burn injury.

From our thematic analysis, more than a third of consent responses attributed personal interest in the study as a reason for participation. Many responses suggested excitement that their buccal samples might reveal why people recover differently from injury. Communicating the use of novel research designs and processes to study candidates may be considered to increase research participation and not seen as an additional burden if well presented. Other studies mention the important role that hospital and research staff play as part of a support system for people living with burn injury, and this is affirmed by the six responses that credit relationships with staff as a reason for their participation in our feasibility study [36]. The nine responses that indicated ease of participation as a reason for consenting are a reminder of the burden people with burn injury face in hospitals and during their recoveries and place a premium on designing research that minimizes additional burden on potential research participants. Privacy was a major concern for those who elected not to participate. Individual genetic information is protected by HIPAA and the 2008 passage of the Genetic Information Nondiscrimination Act (GINA) [39]. Additionally, initiatives such as the Findable, Accessible Interoperable and Reusable (FAIR) Data Principles define good practices in data sharing between research groups [40]. Despite such measures, most patients who refused participation in our genomic study cited privacy concerns, emphasizing the sensitive nature of genetic data [38,41].

As mentioned previously, a logical future step for a project like ours is the use of whole-genome sequencing. Our methods up to DNA isolation and storage would not need to be changed. Whole-genome sequencing would likely need to be performed by a third-party laboratory. Though the cost of sequencing has continued to decrease, it remains significantly more expensive than performing an SNP analysis like we have performed [42]. Isolated DNA can be stored frozen for several years, and the amount of DNA isolated can be aliquoted for use in several projects. As the costs of performing whole-genome sequencing continue to decrease, sample collection and analyses can be reasonably carried out without issue. This project would ultimately require the storage of genomic data in a repository in accordance with the NIH Genomic Data Sharing Policy [43]. The use of an established repository would ensure patient privacy, reliable data storage, and accessibility of our data.

## 5. Conclusions

Our correlation of HRQoL data with COMT variants confirms that genetic data can be integrated into a longitudinal outcomes database. We successfully developed standard operating procedures for genetic sample collection and storage, DNA isolation, and data integration into an existing database, and we demonstrated the feasibility of conducting a multicenter collaborative study using a centralized lab location. Our feasibility study also confirmed that it is possible to correlate genetic mutations, such as COMT allele variation, with commonly used patient-reported outcome measures. In future iterations of such projects, we advocate for a more comprehensive database such as whole-genome sequencing as a means of identifying novel biomarkers that facilitate personalized medicine.

## Figures and Tables

**Table 1:** Quantitative swab collection data.

	Count	DNA Concentration (Median {IQR})	Range
Total	126	67.05 {26.175–179.225}	0–1211.8
Site A	78	103.3 {49.8–292}	2.14–1211.8
Site B	21	30.2 {18.8–58.2}	0–277.8
Site C	27	28.5 {14.4–61.8}	0–181.1
DNA sample transported by mail			
No	68	169.7 {68.025–347.525}	0–1211.8
Yes	58	33.45 {14.5–77.55}	0–277.8
COMT PCR result			
Successful PCR	111	79.5 {33.45–195.7}	4.02–1211.8
Failed PCR	15	8.2 {1.97–25.2}	0–79.7

Concentrations are reported as ng/μL (50 μL total). “DNA sample transported by mail” includes Site A samples collected by participants at home and all swabs from Sites B and C refer to any swabs that traveled by mail to the BMS lab. IQR = interquartile range. COMT = catechol-O-methyltransferase.

**Table 2:** Distribution of COMT SNPs.

	Total	AA “Worrier”	AG Variant	GG “Warrior”
All	111	34	46	31
Site A	75	23	29	23
Site B	15	5	9	1
Site C	21	6	8	7

COMT = catechol-O-methyltransferase. SNP = single nucleotide polymorphism.

**Table 3:** Comparison of COMT genotype to demographics and injury characteristics.

	COMT Genotype			
	AA (n = 34)	AG (n = 46)	GG (n = 31)	*p*-Value
Sex				0.234
Male	27 (36%)	28 (37%)	20 (27%)	
Female	7 (20%)	17 (49%)	11 (31%)	
Age (median {IQR})	47 {36, 58}	48 {34, 64}	46 {29, 61}	0.6968
Race (%)				0.801
White	32 (33%)	38 (39%)	27 (28%)	
Black	1 (33%)	1 (33%)	1 (33%)	
Asian	0 (0%)	1 (100%)	0 (0%)	
American Indian/Alaskan Native	0 (0%)	2 (67%)	1 (33%)	
Native Hawaiian or Other Pacific Islander	0 (0%)	1 (50%)	1 (50%)	
Other	0 (0%)	1 (100%)	0 (0%)	
Ethnicity (%)				0.386
Hispanic/Latino	4 (24%)	6 (35%)	7 (41%)	
Non-Hispanic/Latino	29 (33%)	38 (43%)	22 (25%)	
Burn Size (TBSA) (median {IQR})	10 {6, 22}	18 {6, 37}	13.5 {3, 28}	0.2322
Etiology (%)				0.289
Fire/Flame	24 (32%)	32 (43%)	18 (24%)	
Scald	1 (50%)	0 (0%)	1 (50%)	
Contact	0 (0%)	4 (57%)	3 (43%)	
Grease	4 (27%)	6 (40%)	5 (33%)	
Tar	0 (0%)	0 (0%)	2 (100%)	
Chemical	1 (50%)	1 (50%)	0 (0%)	
Electricity	2 (50%)	1 (25%)	1 (25%)	
Flash	2 (100%)	0 (0%)	0 (0%)	

COMT = catechol-O-methyltransferase. IQR = interquartile range. TBSA = total body surface area.

**Table 4:** Comparison of COMT genotype to patient outcomes.

	COMT Genotype			
	AA	AG	GG	*p*-Value
Outcomes				
VR-12 MCS Preburn	57 {40, 61}	59 {52, 62}	58 {48, 63}	0.49
VR-12 MCS 6-month	55 {45, 59}	54 {43, 59}	57 {45, 63}	0.28
VR-12 PCS Preburn	53 {49, 56}	53 {47, 56}	55 {44, 56}	0.97
VR-12 PCS 6-month	44 {36, 53}	45 {32, 55}	46 {41, 54}	0.88
PTGI 6-month	33 {17, 44}	23 {19, 41}	15 {10, 33}	0.24
Pain Interference (PI) Scores				
PROMIS-29 PI Total	52 {41.6, 61.2}	41.6 {41.6, 61.2}	53.9 {41.6, 55.6}	0.93
VR-12 PI Question	2 {1, 3}	2 {1, 3}	2 {1, 3}	0.86

All data are presented as median values {interquartile range}. COMT = catechol-O-methyltransferase. VR = Veterans RAND-12. PTGI = Post-Traumatic Growth Inventory.

**Table 5:** Thematic analysis of reasons for consenting/declining buccal swab collection.

Theme (n)	Example
Consenting participants (80)	
Altruism (38)	“Any way I can give back—I am willing”
Relationship with burn center staff (6)	“You guys are so great here at Harborview, it gets me emotional just thinking about it”
Interest/enthusiasm (27)	“I find this [research] really interesting”
Ease of participation (9)	“If I’m not being poked with a needle then I’m happy to”
Refusals (26)	
Privacy (14)	“I don’t want to share my genetic info”
Lack of interest (9)	“Not interested”
Other (3)	“If I don’t get the results, I don’t want to participate”In response to study procedures: “Sounds pretty weird”Concerns about COVID susceptibility

All patients who met study criteria were approached for participation and were asked why those chose or declined to participate. Examples are real quotes representative of themes designated by the thematic analysis of responses.

## Data Availability

The Burn Injury Model System National Database is a prospective, longitudinal, and multicenter research data repository that contains measures of functional and psychosocial outcomes following burns. The data are free and publicly available at https://burndata.washington.edu/.
